# High-specificity synthesis of novel monomers by remodeled alcohol hydroxylase

**DOI:** 10.1186/s12896-016-0291-8

**Published:** 2016-08-24

**Authors:** Yanning Zheng, Lingling Li, Qiang Liu, Haibo Zhang, Yujin Cao, Mo Xian, Huizhou Liu

**Affiliations:** 1 CAS Key Laboratory of Biobased Materials, Qingdao Institute of Bioenergy and Bioprocess Technology, Chinese Academy of Sciences, No.189 Songling Road, Laoshan District, Qingdao, 266101 China; 2 College of Food Science, Sichuan Agricultural University, Yaan, 625014 China

**Keywords:** P450_BM3_, Alcohol hydroxylation, *Escherichia coli*, Diols, 1,7-decanediol, Regiospecificity

## Abstract

**Background:**

Diols are important monomers for the production of plastics and polyurethanes, which are widely used in our daily life. The medium-chain diols with one hydroxyl group at its subterminal end are able to confer more flexibility upon the synthesized materials. But unfortunately, this type of diols has not been synthesized so far. The strong need for advanced materials impelled us to develop a new strategy for the production of these novel diols. In this study, we use the remodeled P450_BM3_ for high-specificity production of 1,7-decanediol.

**Results:**

The native P450_BM3_ was capable of converting medium-chain alcohols into corresponding α, ω1-, α, ω2- and α, ω3-diols, with each of them accounting for about one third of the total diols, but it exhibited a little or no activity on the short-chain alcohols. Greatly improved regiospecificity of alcohol hydroxylation was obtained by laboratory evolution of P450_BM3_. After substitution of 12 amino acid residues (J2-F87A), the ratio of 1,7-decanediol (ω-3 hydroxylation) to total decanediols increased to 86.8 % from 34.0 %. Structure modeling and site-directed mutagenesis demonstrated that the heme end residues such as Ala^78^, Phe^87^ and Arg^255^ play a key role in controlling the regioselectivity of the alcohol hydroxylation, while the residues at the mouth of substrate binding site is not responsible for the regioselectivity.

**Conclusions:**

Herein we employ an engineered P450_BM3_ for the first time to enable the high-specificity biosynthesis of 1,7-decanediol, which is a promising monomer for the development of advanced materials. Several key amino acid residues that control the regioselectivity of alcohol hydroxylation were identified, providing some new insights into how to improve the regiospecificity of alcohol hydroxylation. This report not only provides a good strategy for the biosynthesis of 1,7-decanediol, but also gives a promising approach for the production of other useful diols.

**Electronic supplementary material:**

The online version of this article (doi:10.1186/s12896-016-0291-8) contains supplementary material, which is available to authorized users.

## Background

Diols are of great importance in the manufacture of plastics and polyurethanes, which have molded our society in many ways that make our life much easier [1]. The biosynthesis of short-chain diols such as 1,3-propanediol and 1,4-butanediol has been well developed [2–5], while the long-chain α, ω-diols such as 1,14-tetradecanediol and 1,16-hexadecanediol can be made by the catalytic hydrogenation of long-chain dicarboxylate esters. The medium-chain diols with one subterminal hydroxyl group, for example, 1,7-decanediol, which confer more flexibility upon the synthesized materials, are promising monomers for the synthesis of polymers with better properties. But unfortunately, this type of diols has not yet been synthesized with either chemical or biological method. The strong market demand for new polymers drives us to develop a strategy for the synthesis of these diols.

**Fig. 1:**
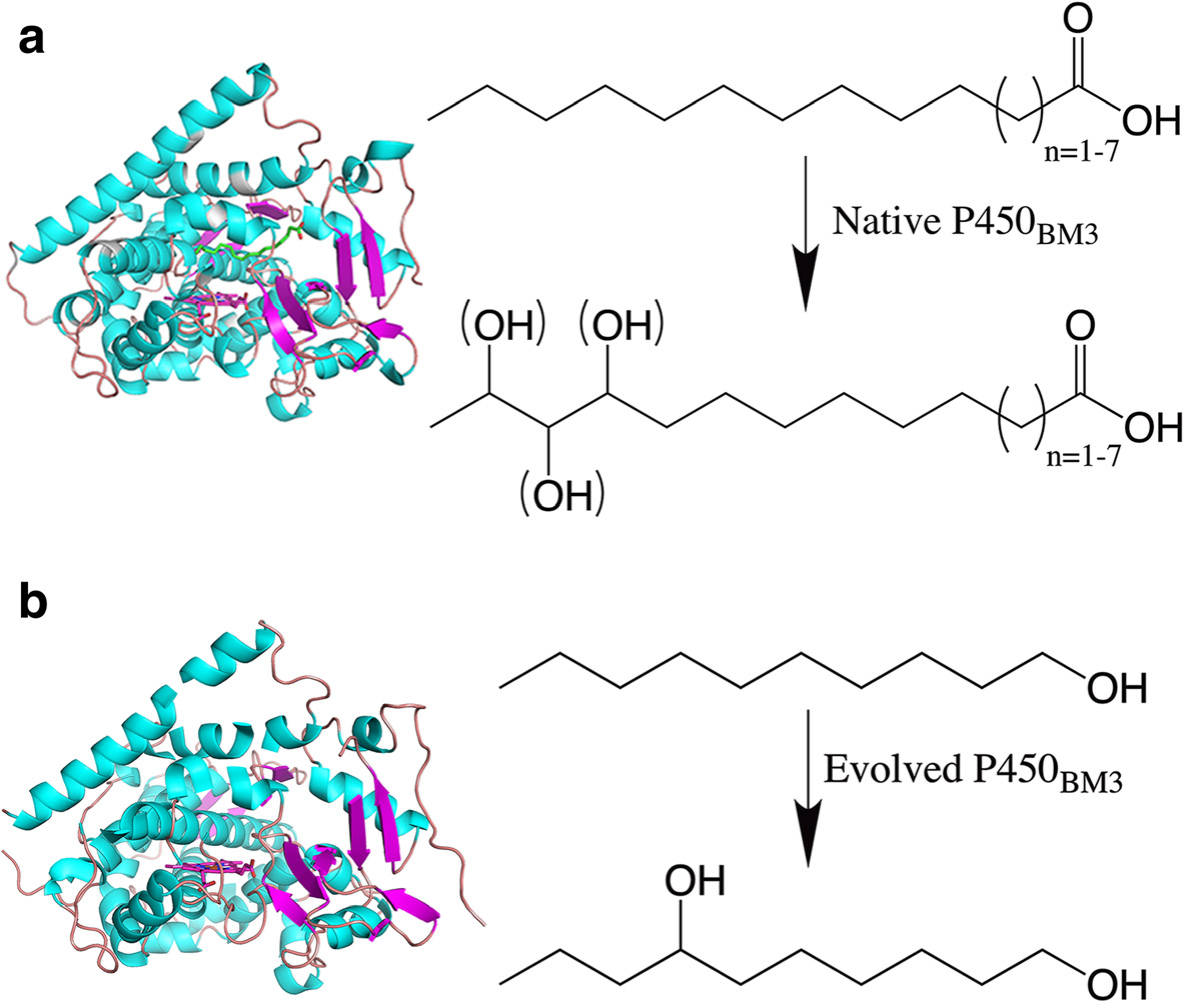
Schematic representation of the substrate specificities and regioselectivities of the native P450_BM3_ and the remodeled P450_BM3_. The native P450_BM3_ has a substrate preference for C12-C18 fatty acids and evenly oxidizes the ω-1, ω-2 and ω-3 carbons of the fatty acid substrates (**a**), while the remodeled P450_BM3_ exhibits a greatly improved regiospecificity of decanol hydroxylation, with 1,7-decanediol as the dominant product (**b**)

To obtain the carbon backbones, we paid attention to the straight-chain fatty alcohols, whose biosynthesis has been well developed in recent years [6–8]. To finally get the desired diols, an additional hydroxyl group needs to be added to the subterminal carbon of the monohydric alcohols. But it is too difficult for the inorganic catalysts to catalyze the oxidation of a specific subterminal carbon of the monohydric alcohols, as each of the subterminal carbons almost has the same chemical contexts, and the inorganic catalysts usually exhibit poor selectivity. So the biocatalyst was considered as a priority. In seeking such a biocatalyst for the conversion of monohydric alcohols to corresponding diols, P450_BM3_ seems to be a promising enzyme, which naturally catalyzes the conversion of long-chain fatty acids to corresponding hydroxyfatty acids [9, 10]. P450_BM3_ is highly soluble in cytosolic environment, and has high catalytic rates and expression level in engineered *E. coli*, which is the most widely used host for the bioproduction of chemicals [11, 12]. These advantages make it an ideal biocatalyst for biotechnological application. And more importantly, its variants have shown to be able to utilize a wide range of other substrates. The P450_BM3_ variant 35-E11, whose 17 amino acid residues were substituted, was found to be capable of converting ethane to ethanol [11], and a series of P450_BM3_ variants were reported the improved activities on non-natural substrates naphthalene, pentane, p-cymene and propylbenzene [13]. These findings suggest that P450_BM3_ has stunning flexibility in substrate preference. In addition, protein engineering has been widely used as a strategy in the biological production of chemicals [14, 15]. Therefore, an improved regioselectivity for alcohol hydroxylation at ω-3 position can be expected by remodeled P450_BM3_ (Fig. 1).

In this study, we are reporting a strategy for high-specificity synthesis of α, ω3-diols from renewable medium-chain fatty alcohols. To improve the regioselectivity for alcohol hydroxylation at ω-3 position, we generated a series of P450_BM3_ variants by laboratory evolution. We also identified several key amino acid residues that control the regioselectivity of alcohol hydroxylation, and discussed the reason why these residues play a key role in determining the regioselectivity by structural analysis.

## Results and discussion

### Substrate specificity and regioselectivity of P450_BM3_

**Fig. 2:**
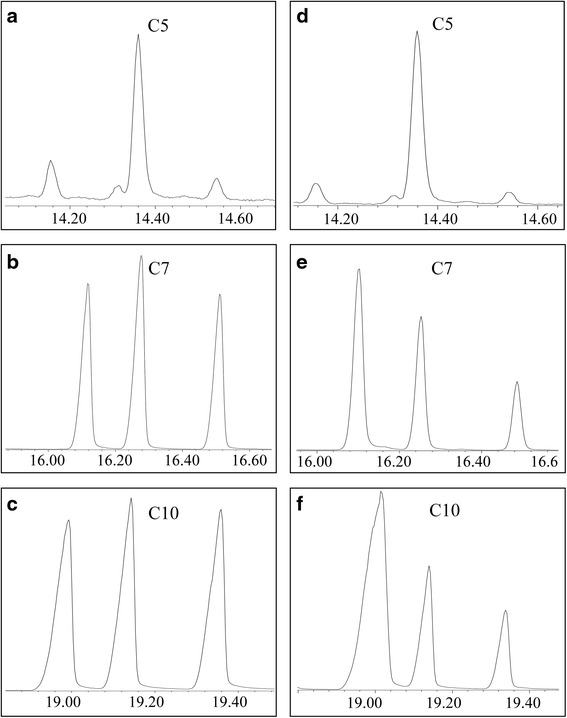
GC-MS analysis of diols in the cultures. When pentanol was used as substrate, both BM3 and BM3J produced 1,4-pentanediol as the only product (**a**, **d**). When heptanol and decanol were used as substrates, BM3J produced much more 1,4-heptanediol (**b**, **e**) and 1,7-decanediol (**c**, **f**) than BM3, respectively

**Fig. 3:**
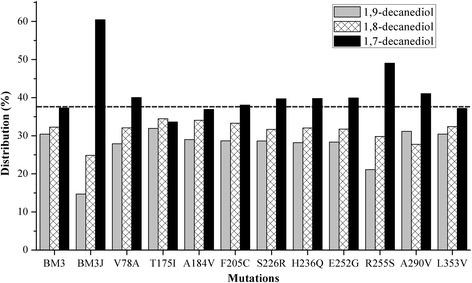
Regiospecificities of the native P450_BM3_, P450_BM3J_ and corresponding 10 single mutants of P450_BM3J_. Decanol was used as the substrate, and 1,7-, 1,8- and 1,9-decanediols were analyzed by GC-MS. The ratio of 1,7-decanediol to total decanediols increased from 34.0 % in BM3 to ~50 % in R255S, suggesting the increased ratio of 1,7-decanediol to total decanediols in BM3J is mainly attributed to the substitution of Arg^255^ to Ser^255^. The other 9 amino acid substitutions may have a combined effect on the constriction of the substrate-binding channel and the change of the substrate orientation

The P450_BM3_ was chosen as the starting enzyme for the conversion of alcohols to diols. So the engineered *E. coli* that overexpresses the native P450_BM3_ was constructed as the biocatalyst to test P450_BM3_’s ability of alcohol hydroxylation. The heptanol and decanol were firstly used as the substrates for testing the activity of P450_BM3_, as the medium chain diols are of more interests for the development of new polymers. P450_BM3_ exhibited high activities towards these substrates and produced almost an equivalent amount of α, ω1-, α, ω2- and α, ω3-diols, exhibiting the same regioselectivity as its native fatty acid substrates (Figs. 2 and 3). But when using pentanol as the substrate, only a tiny amount of 1,4-pentanediol was obtained (Fig. 2), suggesting P450_BM3_ has a different regioselectivity for short-chain alcohols. No activity was observed when using propanol and butanol as the substrates.

As the typical substrates of P450_BM3_ are long-chain fatty acids [16], it is not surprising that the short-chain fatty alcohols are poor substrates for P450_BM3_. Arg^47^ and Tyr^51^ were thought to interact with the carboxylate group of the fatty acid substrates [17, 18]. Our finding that P450_BM3_ is also capable of utilizing fatty alcohols demonstrates that the interaction between Arg^47^/Tyr^51^ and carboxylate group is not so strong, and Arg^47^/Tyr^51^ is not involved in determining the substrate specificity of P450_BM3_. If Arg^47^/Tyr^51^ is responsible for stabilizing the carboxylate group of the fatty acid substrates, the carboxylate group needs to be recognized by the two amino acid residues, and P450_BM3_ will not be able to oxidize those hydrocarbons without carboxylate group. Therefore, the size of the substrate-binding pocket plays an important role in determining the substrate specificity of P450_BM3_, given that the P450_BM3_ has no activity towards short-chain alcohols and alkanes [19].

### Substrate specificity and regioselectivity of P450_BM3J_

For the high-specificity production of the α, ω3-diols, the native P450_BM3_ needs to be modified to change its regioselectivity for alcohol hydroxylation. The regioselectivity is connected with the substrate orientation, which could be changed when the substrate channel of P450_BM3_ is constrained. It was found that a P450_BM3_ variant (P450_BM3J_), which contains 10 amino acid substitutions (V78A, T175I, A184V, F205C, S226R, H236Q E252G, R255S, A290V, L353V) with respect to the native P450_BM3_, changed its substrate preferences for shorter alkanes (C4-C8) when compared with the native P450_BM3_, which exhibited a substrate preference for Cn > 8 alkanes [19]. It could be expected that P450_BM3J_ will have a higher priority for ω3 hydroxylation when using a longer-chain alcohol substrate. So we generated another engineered *E. coli* BM3J that overexpressed P450_BM3J_. The same as BM3, BM3J had no activity on propanol and butanol, and exhibited a low activity on pentanol, with 1,4-pentanediol as the only product (Fig. 2). But when using heptanol and decanol as substrates, BM3J produced more 1,4-heptanediol and 1,7-decanediol than BM3, with 1,4-heptanediol and 1,7-decanediol accounting for 50.1 and 64.5 % of total heptanediols and decanediols, respectively (Figs. 2 and 3).

**Fig. 4:**
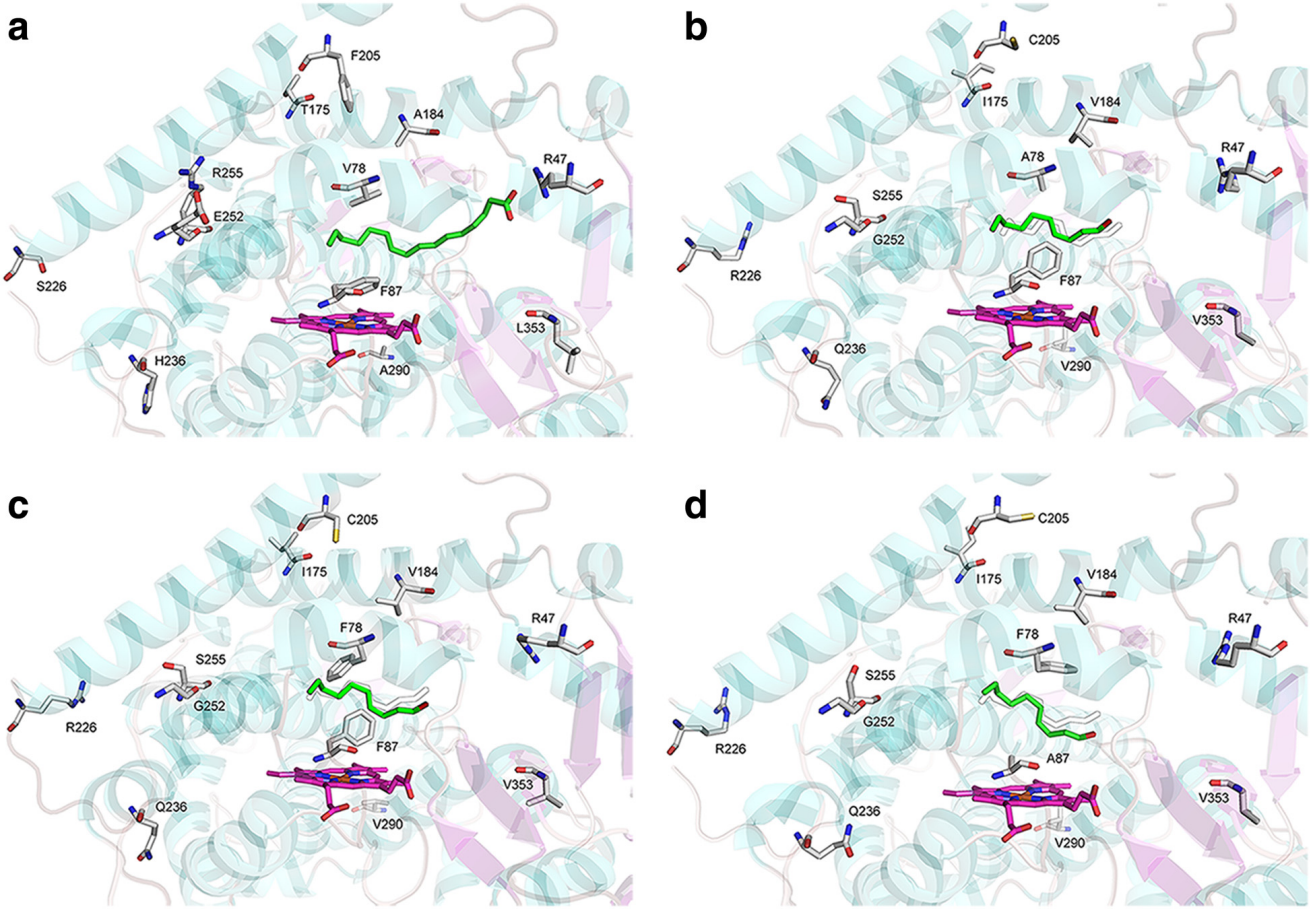
Three-dimensional structures of P450_BM3_ and its variants. P450_BM3_ heme domain with palmitoleic acid bound was obtained from 1FAG (**a**). SWISS-MODEL derived homology models of P450_BM3_ variant structures, using 1FAG as the template (**b**, **c**, **d**). The amino acid residues involved in laboratory evolution are depicted in stick symbols. B, P450_BM3J_; C, P450_J2_; D, P450_J2-F87A_

To examine if the increased ω-3 hydroxylation is mainly attributed to a specific amino acid substitution, we made 10 single mutants that correspond to the 10 amino acid substitutions of P450_BM3J_, namely, V78A, T175I, A184V, F205C, S226R, H236Q, E252G, R255S, A290V and L353V. The ratio of 1,7-decanediol to total decanediols increased from 34.0 % in BM3 to ~50 % in R255S (Fig. 3), suggesting the increased ratio of 1,7-decanediol to total decanediols in BM3J is mainly attributed to the substitution of Arg^255^ to Ser^255^, which allows the ω terminal carbon of the decanol substrate to move a little closer to the Ser^255^, and in the meanwhile to move a little farther away from the heme. This movement makes the ω-1 carbon deviate from its best position for hydroxylation, and thus decreases the efficiency of ω-1 hydroxylation. The other 9 amino acid substitutions may have a combined effect on the constriction of the substrate-binding channel and the change of the substrate orientation (Fig. 4b).

### The effect of heme end residues on the regioselectivity of P450_BM3J_

**Fig. 5:**
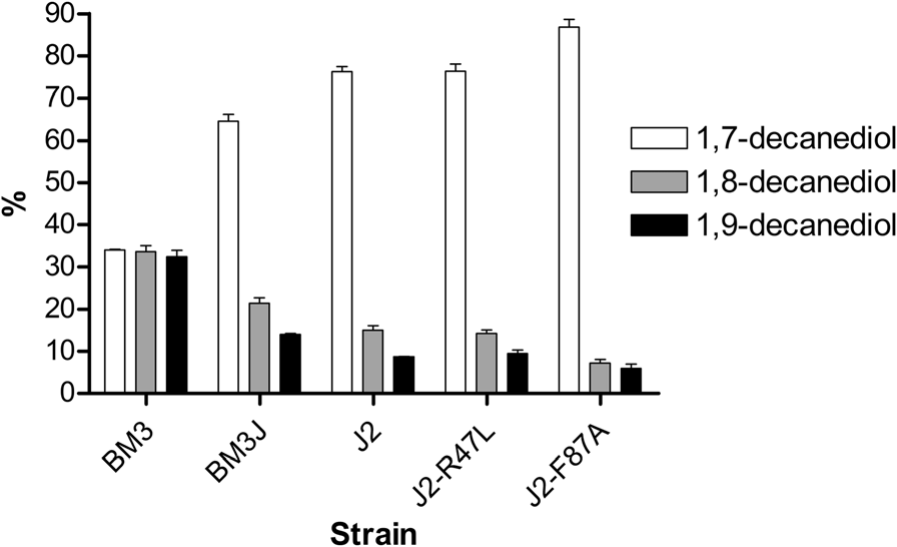
Regioselectivities of native P450_BM3_ and its variants for decanol. Decanol was used as substrate, and product decanediols were analyzed by GC-MS. 1,7-, 1,8- and 1,9-decanediols represent ω-3, ω-2 and ω-1 hydroxylation, respectively. BM3 expresses the native P450_BM3_, while BM3J, J2, J2-R47L and J2-F87A express different P450_BM3_ variants

To further improve the regioselectivity of P450_BM3J_, we need to further constrict the substrate-binding channel. The Val^78^ in P450_BM3_ is located near the ω terminus of fatty acid substrates, so it may be vital in controlling the substrate orientation. Fatty alcohol substrates should have a quite similar substrate orientation in the active site to the fatty acid substrates, which can be reflected by the regioselectivity for heptanol and decanol hydroxylation. The substitution of Val^78^ in P450_BM3_ to Ala^78^ in P450_V78A_ nearly does not change the regioselectivity for decanol, as alanine has a similar property to valine. But when the Ala^78^ in P450_BM3J_ was substituted to Phe^78^, yielding a variant J2, the ratio of 1, 7-decanediol (ω-3 hydroxylation) to total decanediols further increased to 76.3 % from 64.5 % in BM3J (Fig. 5). The phenyl group of Phe^78^ creates a narrower space between Phe^78^ and the heme. This conformational change forced the carbon chain bend, made the ω, ω-1 and ω-2 terminal carbons move farther away from the heme, and finally resulted in the decrease of undesired ω-1 and ω-2 hydroxylation (Fig. 4c). Therefore, the residues located around the heme end of the substrate-binding channel indeed play more important roles in determining the regioselectivity of the alcohol hydroxylases for medium-chain alcohol substrates.

To test this hypothesis, two residues, Arg^47^ and Phe^87^, were chosen for further modification. Arg^47^ is located at the mouth of substrate binding site and its guanidinium group is thought to provide an important ion-pair interaction with the carboxylate group of the fatty acid substrates [20], while Phe^87^ is located above the heme and is known to be an important factor in determining the regioselectivity of substrate hydroxylation [21]. The substitution of Arg^47^ to Leu^47^ was found to increase the hydroxylase activity towards pentane and propylbenzene [13]. The R47L mutation was then incorporated into the variant J2, generating a new variant J2-R47L. As expected, the regiospecificity profile of J2-R47L was nearly the same as that of J2 (Fig. 5), demonstrating the substitution of Arg^47^ to Leu^47^ did not change the substrate orientation and had little connection with the regioselectivity for alcohol substrates. But when the Phe^87^ was substituted to Ala^87^ (J2-F87A), the ratio of 1,7-decandediol to total decanediols further increased to 86.8 % (Fig. 5). The substitution of Phe^87^ to Ala^87^ released the space for the α-terminus of decanol to move towards the heme, and in the meanwhile made the benzene ring of Phe^78^ rotate away from the ω terminus of decanol (Fig. 4d). This incident allowed the ω, ω-1 and ω-2 terminal carbons of decanol to further move away from the heme, leading to the increased distribution of 1,7-decandediol. The heme end residues are responsible for contraction or expansion of the hydrophobic pocket, so they can affect the alcohol orientation in the substrate-binding channel and finally control the regioselectivity of the alcohol hydroxylation.

## Conclusions

The engineering strategy described above inaugurates a new realm for the high-specificity production of 1,7-decanediol, which is a promising monomer for the development of advanced materials. The desired 1,7-decanediol was finally produced, being the first successful report on the biosynthesis of diols with one hydroxyl group at the subterminus. The regiospecificity of alcohol hydroxylation was greatly improved by laboratory evolution. Conservative structural models of the P450_BM3_ variants demonstrate that the heme end residues in the substrate-binding channel play a key role in determining the regioselectivity for medium-chain alcohols. This study not only provides a good strategy for the biosynthesis of 1,7-decanediol, but also gives a promising approach for the production of other useful diols. More and more advanced materials can be expected once these new diol monomers are available.

## Methods

### Plasmid construction

The *P450*_*BM3*_ gene was amplified from genomic DNA of *Bacillus megaterium* ATCC 14581 (NZ_CP009920) with the primer set BM3-NcoF and BM3-BamHR. The PCR product digested with NcoI and BamHI was cloned into pCOLADuet-1 (Novagen, Darmstadt, Germany) cut with the same restriction enzymes, creating pLQ12. P450_BM3J_ was generated by introducing 10 amino acid mutations (V78A, T175I, A184V, F205C, S226R, H236Q, E252G, R255S, A290V, L353V) on P450_BM3_ [19]. The codon-optimized 1129 bp necleic acids coding for the N-terminal P450_BM3J_ were chemically synthesized, amplified with the primer set BM3J-NcoF and BM3J-EcoR, and integrated into the pCOLADuet-1, creating the plasmid pZZ1-up. The other part of the *P450*_*BM3J*_ gene, which is the same as that of *P450*_*BM3*_ gene, was amplified from genomic DNA of *B. megaterium* (ATCC 14581) with the primer set BM3J-EcoF and BM3J-NotR. The PCR product digested with EcoRI and NotI was cloned into pZZ1-up cut with the same restriction enzymes, creating pZZ1. The whole *P450*_*BM3J*_ gene sequence is shown in [Additional file 1: Figure S1].

### Structure modeling and site-directed mutagenesis

The models of the P450 variant structures were built on a public server Swiss-Model, using 1FAG as the template [22–24]. A method based on the amplification of the entire plasmid using primers that include the desired changes was employed for the site-directed mutagenesis [25]. All the plasmids and strains used in this work are listed in Table 1, and the oligonucleotide primers are given in Table 2.

**Table 1:** Bacterial strains and plasmids used in this study

Plasmid or strain	Relevant genotype or description	Reference
Plasmids		
pCOLADuet-1	ColA origin; Kan^R^; P_T7_	Novagen
pLQ12	ColA origin; Kan^R^; P_T7_:: *P450*_*BM3*_	This study
pZZ1	ColA origin; Kan^R^; P_T7_:: *P450*_*BM3J*_	This study
pV78A	ColA origin; Kan^R^; P_T7_:: *P450*_*BM3V78A*_	This study
pT175I	ColA origin; Kan^R^; P_T7_:: *P450*_*BM3T175I*_	This study
pA184V	ColA origin; Kan^R^; P_T7_:: *P450*_*BM3A184V*_	This study
pF205C	ColA origin; Kan^R^; P_T7_:: *P450*_*BM3F205C*_	This study
pS226R	ColA origin; Kan^R^; P_T7_:: *P450*_*BM3S226R*_	This study
pH236Q	ColA origin; Kan^R^; P_T7_:: *P450*_*BM3H236Q*_	This study
pE252G	ColA origin; Kan^R^; P_T7_:: *P450*_*BM3E252G*_	This study
pR255S	ColA origin; Kan^R^; P_T7_:: *P450*_*BM3R255S*_	This study
pA290V	ColA origin; Kan^R^; P_T7_:: *P450*_*BM3A290V*_	This study
pL353V	ColA origin; Kan^R^; P_T7_:: *P450*_*BM3L353V*_	This study
pJ2	ColA origin; Kan^R^; P_T7_:: *P450*_*BM3JA78F*_	This study
pJ2-R47L	ColA origin; Kan^R^; P_T7_:: *P450*_*BM3JA78FR47L*_	This study
pJ2-F87A	ColA origin; Kan^R^; P_T7_:: *P450*_*BM3JA78F87A*_	This study
Strains		
BL21(DE3)	*E. coli B dcm ompT hsdS*(r_B_^−^m_B_^−^) *gal*	Invitrogen
LQ12	BL21(DE3) harboring pLQ12	This study
ZZ1	BL21(DE3) harboring pZZ1	This study
V78A	BL21(DE3) harboring pV78A	This study
T175I	BL21(DE3) harboring pT175I	This study
A184V	BL21(DE3) harboring pA184V	This study
F205C	BL21(DE3) harboring pF205C	This study
S226R	BL21(DE3) harboring pS226R	This study
H236Q	BL21(DE3) harboring pH236Q	This study
E252G	BL21(DE3) harboring pE252G	This study
R255S	BL21(DE3) harboring pR255S	This study
A290V	BL21(DE3) harboring pA290V	This study
L353V	BL21(DE3) harboring pL353V	This study
J2	BL21(DE3) harboring pJ2	This study
J2-R47L	BL21(DE3) harboring pJ2-R47L	This study
J2-F87A	BL21(DE3) harboring pJ2-F87A	This study

**Table 2:** Primers used in this study

Name	Sequence (5′ → 3′)
BM3-NcoF	CTTGCCATGGGCATGACAATTAAAGAAATGCCTCAG
BM3-BamR	CGGGATCCTTACCCAGCCCACACGTCTTTTG
BM3J-NcoF	CATGCCATGGGCATGACAATTAAAGAAATGCCTCAG
BM3J-EcoR	GACGGAATTCTTCCACATCG
BM3J-EcoF	GAAGAATTCCGTCCAGAGCGTTTTG
BM3J-NotR	ATAAGAATGCGGCCGCTTACCCAGCCCACACGTCTTTTG
BM3-V78A-F	GCTTAAATTTGCACGTGATTTTGCAGGAGACGG
BM3-V78A-R	CAAAATCACGTGCAAATTTAAGCGCTTGACTTAAG
BM3-T175I-F	CCATTTATTATCAGTATGGTCCGTGCACTGGATG
BM3-T175I-R	GACCATACTGATAATAAATGGATGAGGCTGATC
BM3-A184V-F	GGATGAAGTAATGAACAAGCTGCAGCGAGC
BM3-A184V-R	CTTGTTCATTACTTCATCCAGTGCACGGACC
BM3-F205C-F	CAAGCGCCAGTGTCAAGAAGATATCAAGGTG
BM3-F205C-R	CTTCTTGACACTGGCGCTTGTTTTCATCATAAG
BM3-S226R-F	CGCAAAGCACGCGGTGAACAAAGCGATG
BM3-S226R-R	GTTCACCGCGTGCTTTGCGATCTGC
BM3-H236Q-F	CGCAGATGCTAAACGGAAAAGATCCAG
BM3-H236Q-R	CCGTTTAGCATCTGCGTTAATAAATCATC
BM3-E252G-F	GATGACGGGAACATTCGCTATCAAATTATTAC
BM3-E252G-R	GCGAATGTTCCCGTCATCAAGCGGCTCACCCG
BM3-R255S-F	GACGAGAACATTAGCTATCAAATTATTACATTC
BM3-R255S-R	GATAGCTAATGTTCTCGTCATCAAGCGGCTCAC
BM3-A290V-F	GTATTACAAAAAGTAGCAGAAGAAGCAGC
BM3-A290V-R	CTTCTGCTACTTTTTGTAATACATGTGG
BM3-L353V-F	GCGACGAAGTAATGGTTCTGATTCCTCAGC
BM3-L353V-R	GAACCATTACTTCGTCGCCTTTTTCTAAAGG
BM3J-A78F-F	GCGCTGAAATTTTTCCGTGATTTTGCAGGTGACGG
BM3J-A78F-R	CAAAATCACGGAAAAATTTCAGCGCTTGACTTAAG
J2-R47L-F	GCGCCTGGTCTGGTAACGCGCTACTTATCAAG
J2-R47L-R	CGCGTTACCAGACCAGGCGCCTCGAATTTAAAG
J2-F87A-F	GACGGGTTGGCTACAAGCTGGACGCATG
J2-F87A-R	GCTTGTAGCCAACCCGTCACCTGCAAAATC

Underlines indicate restriction enzyme sites

### Bacterial strains, media and growth conditions

The bacterial strains used in this study are listed in Table S1. *E. coli* BL21(DE3) (Invitrogen, Carlsbad, CA) was used as the host to overproduce proteins. During strain construction, cultures were grown aerobically at 37 °C in LB medium (10 g/L tryptone, 10 g/L NaCl, and 5 g/L yeast extract). Kanamycin (50 mg/L) was added if necessary. For initial protein over-production in shake flasks, cultures were firstly grown in LB medium containing 50 mg/L kanamycin, then induced with 0.5 mM isopropyl β-D-thiogalactoside (IPTG), next incubated at 30 °C for 10 h, and finally harvested by centrifugation. The cell catalysts obtained from 50 ml cultures were transferred into 25 ml M9 medium (37.8 g/L Na_2_HPO_4_ · 12H_2_O, 7.5 g/L KH_2_PO_4_, 1 g/L NH_4_Cl, 0.5 g/L NaCl, 4 mM MgSO4) supplemented with 50 mg/L kanamycin, 0.25 mM IPTG and 5 mM alcohols, and incubated at 30 °C for 48 h.

### Analysis of diols by GC-MS

Different diols produced by the engineered strains were identified by gas chromatography–mass spectrometry (GC–MS). These diols were isolated by ethyl acetate extraction. After concentrated by a rotary evaporator and redissolved with ethanol, 1 μl sample was injected for GC-MS analysis. The system consisted of model 7890A network GC system (Agilent Technologies) and a model 5975C network mass selective detector (Agilent Technologies, Santa Clara, CA). A HP-INNOWAX capillary column (30 m × 0.25 mm; 0.25 μm film thickness; Agilent Technologies) was used, with helium as the carrier gas. The following oven temperature program was carried out: 50 °C for 2 min, increase of 10 °C/min to 240 °C, 240 °C for 5 min. The injector was maintained at 250 °C.

## Additional file

Additional file 1: Figure S1. Nucleotide sequence of P450_BM3J_. (DOCX 30 kb)

## Abbreviations

*E. coli*: *Escherichia coli*
GC-MS: Gas chromatography-mass spectrometry
IPTG: Lsopropyl β-D-thiogalactoside
Kan: Kanamycin
PCR: Polymerase chain reaction.

## References

[1] Hu S, Li Y (2014). Two-step sequential liquefaction of lignocellulosic biomass by crude glycerol for the production of polyols and polyurethane foams. Bioresour Technol.

[2] Jiang Y, Liu W, Zou H, Cheng T, Tian N, Xian M (2014). Microbial production of short chain diols. Microb Cell Fact.

[3] Nakamura CE, Whited GM (2003). Metabolic engineering for the microbial production of 1,3-propanediol. Curr Opin Biotech.

[4] Yim H, Haselbeck R, Niu W, Pujol-Baxley C, Burgard A, Boldt J, Khandurina J, Trawick JD, Osterhout RE, Stephen R (2011). Metabolic engineering of *Escherichia coli* for direct production of 1,4-butanediol. Nat Chem Bio.

[5] Moscoviz R, Trably E, Bernet N (2016). Consistent 1,3-propanediol production from glycerol in mixed culture fermentation over a wide range of pH. Biotechnol Biofuels.

[6] Steen EJ, Kang Y, Bokinsky G, Hu Z, Schirmer A, McClure A, Del Cardayre SB, Keasling JD (2010). Microbial production of fatty-acid-derived fuels and chemicals from plant biomass. Nature.

[7] Zheng YN, Li LL, Liu Q, Yang JM, Wang XW, Liu W, Xu X, Liu H, Zhao G, Xian M (2012). Optimization of fatty alcohol biosynthesis pathway for selectively enhanced production of C12/14 and C16/18 fatty alcohols in engineered *Escherichia coli*. Microb Cell Fact.

[8] Mattam AJ, Yazdani SS (2013). Engineering *E. Coli* strain for conversion of short chain fatty acids to bioalcohols. Biotechnol Biofuels.

[9] Schneider S, Wubbolts MG, Sanglard D, Witholt B (1998). Biocatalyst engineering by assembly of fatty acid transport and oxidation activities for In vivo application of cytochrome P-450_BM-3_ monooxygenase. App Enviro Microbiol.

[10] Cao Y, Cheng T, Zhao G, Niu W, Guo J, Xian M, Liu H (2016). Metabolic engineering of *Escherichia coli* for the production of hydroxy fatty acids from glucose. BMC Biotechnol.

[11] Meinhold P, Peters MW, Chen MM, Takahashi K, Arnold FH (2005). Direct conversion of ethane to ethanol by engineered cytochrome P450 BM3. Chembiochem.

[12] Chen YY, Shen HJ, Cui YY, Chen SG, Weng ZM, Zhao M, Liu JZ (2013). Chromosomal evolution of *Escherichia coli* for the efficient production of lycopene. BMC Biotechnol.

[13] Whitehouse CJ, Bell SG, Tufton HG, Kenny RJ, Ogilvie LC, Wong LL (2008). Evolved CYP102A1 (P450_BM3_) variants oxidise a range of non-natural substrates and offer new selectivity options. Chem Commun.

[14] Joshi S, Satyanarayana T (2015). In vitro engineering of microbial enzymes with multifarious applications: prospects and perspectives. Bioresour Technol.

[15] Zheng H, Wang X, Yomano LP, Geddes RD, Shanmugam KT, Ingram LO (2013). Improving *Escherichia coli* FucO for furfural tolerance by saturation mutagenesis of individual amino acid positions. App Enviro Microbiol.

[16] Lentz O, Urlacher V, Schmid RD (2004). Substrate specificity of native and mutated cytochrome P450 (CYP102A3) from *Bacillus subtilis*. J Biotechnol.

[17] Li H, Poulos TL (1997). The structure of the cytochrome p450BM-3 haem domain complexed with the fatty acid substrate, palmitoleic acid. Nat Struct Biol.

[18] Oliver CF, Modi S, Primrose WU, Lian LY, Roberts GC (1997). Engineering the substrate specificity of *Bacillus megaterium* cytochrome P-450 BM3: hydroxylation of alkyl trimethylammonium compounds. Biochem J.

[19] Fasan R, Meharenna YT, Snow CD, Poulos TL, Arnold FH (2008). Evolutionary history of a specialized P450 propane monooxygenase. J Mol Biol.

[20] Noble MA, Miles CS, Chapman SK, Lysek DA, MacKay AC, Reid GA, Hanzlik RP, Munro AW (1999). Roles of key active-site residues in flavocytochrome P450 BM3. Biochem J.

[21] Graham-Lorence S, Truan G, Peterson JA, Falck JR, Wei S, Helvig C, Capdevila JH (1997). An active site substitution, F87V, converts cytochrome P450 BM-3 into a regio- and stereoselective (14S,15R)-arachidonic acid epoxygenase. J Biol Chem.

[22] Biasini M, Bienert S, Waterhouse A, Arnold K, Studer G, Schmidt T, Kiefer F, Cassarino TG, Bertoni M, Bordoli L, Schwede T (2014). SWISS-MODEL: modelling protein tertiary and quaternary structure using evolutionary information. Nucleic Acids Res.

[23] Arnold K, Bordoli L, Kopp J, Schwede T (2006). The SWISS-MODEL workspace: a web-based environment for protein structure homology modelling. Bioinformatics.

[24] Kiefer F, Arnold K, Kunzli M, Bordoli L, Schwede T (2009). The SWISS-MODEL Repository and associated resources. Nucleic Acids Res.

[25] Hemsley A, Arnheim N, Toney MD, Cortopassi G, Galas DJ (1989). A simple method for site-directed mutagenesis using the polymerase chain reaction. Nucleic Acids Res.

