# Supplementary material for: High-specificity synthesis of novel monomers by remodeled alcohol hydroxylase
Source: BMC Biotechnol. 2016 Aug 24;16(1):61. doi: 10.1186/s12896-016-0291-8 (PMC4995789; doi:10.1186/s12896-016-0291-8)
Supplement: Additional file 1: Figure S1. — Nucleotide sequence of P450BM3J. (DOCX 30 kb) [file 12896_2016_291_MOESM1_ESM.docx]

High-specificity synthesis of novel monomers by remodeled alcohol hydroxylase

Yanning Zheng ^1^**^‡^**, Lingling Li ^1,2^**^‡^**, Qiang Liu ^1,2^, Haibo Zhang ^1^, Yujin Cao ^1^, Mo Xian ^1*^, Huizhou Liu ^1*^

^1^ Qingdao Institute of Bioenergy and Bioprocess Technology, Chinese Academy of Sciences, Qingdao, 266101 China

^2^ College of Food Science, Sichuan Agricultural University, Yaan, 625014, China

*Correspondences: xianmo@qibebt.ac.cn (Mo Xian); liuhz@qibebt.ac.cn (Huizhou Liu)

Mailing address: No.189 Songling Road, Laoshan District, Qingdao, 266101, China

Tel: 86-532-80662766

Fax: 86-532-80662765

**Fig. S1** Nucleotide sequence of P450_BM3J_.

ATGACAATTAAAGAAATGCCTCAGCCAAAAACGTTTGGCGAGCTGAAAAATTTACCGCTGTTGAACACAGATAAACCGGTTCAAGCTCTGATGAAAATTGCGGATGAACTGGGTGAAATCTTTAAATTCGAGGCGCCTGGTCGTGTAACGCGCTACTTATCAAGTCAGCGTCTGATTAAAGAAGCATGCGATGAATCTCGCTTTGATAAAAACTTAAGTCAAGCGCTGAAATTTGCACGTGATTTTGCAGGTGACGGGTTGTTTACAAGCTGGACGCATGAAAAAAATTGGAAAAAAGCGCATAATATCTTACTGCCGAGTTTCAGTCAGCAGGCAATGAAAGGCTATCATGCGATGATGGTTGACATTGCCGTGCAGCTTGTTCAAAAGTGGGAGCGTCTGAATGCAGATGAGCATATTGAAGTACCGGAAGACATGACACGTTTGACGCTGGATACAATTGGTCTTTGCGGCTTTAACTATCGCTTTAACAGCTTTTACCGTGATCAGCCTCATCCATTTATTATTAGTATGGTCCGTGCACTGGATGAAGTAATGAACAAGCTGCAGCGTGCAAATCCAGACGACCCAGCTTATGATGAAAACAAGCGCCAGTGTCAAGAAGACATTAAGGTGATGAACGACCTGGTAGATAAAATTATTGCAGATCGCAAAGCACGCGGTGAACAAAGCGATGATTTGCTGACGCAGATGCTGAACGGCAAAGATCCAGAAACGGGTGAGCCGCTTGATGACGGGAACATTAGCTATCAAATTATTACATTCTTAATTGCGGGTCACGAAACAACCAGTGGTCTGTTATCATTTGCGCTGTATTTCTTGGTGAAAAATCCACATGTATTACAAAAAGTAGCAGAAGAAGCAGCACGCGTTCTGGTAGATCCTGTTCCAAGCTACAAACAAGTCAAACAGCTTAAATATGTCGGCATGGTCTTAAACGAAGCGCTGCGCTTGTGGCCAACTGCTCCTGCGTTTTCCCTGTATGCAAAAGAAGATACGGTGCTTGGCGGTGAATATCCTTTGGAAAAAGGCGACGAAGTAATGGTTCTGATTCCTCAGCTGCACCGTGATAAAACAATTTGGGGAGACGATGTGGAAGAATTCCGTCCAGAGCGTTTTGAAAATCCAAGTGCGATTCCGCAGCATGCGTTTAAACCGTTTGGAAACGGTCAGCGTGCGTGTATCGGTCAGCAGTTCGCTCTTCATGAAGCAACGCTGGTACTTGGTATGATGCTAAAACACTTTGACTTTGAAGATCATACAAACTACGAGCTGGATATTAAAGAAACTTTAACGTTAAAACCTGAAGGCTTTGTGGTAAAAGCAAAATCGAAAAAAATTCCGCTTGGCGGTATTCCTTCACCTAGCACTGAACAGTCTGCTAAAAAAGTACGCAAAAAGGCAGAAAACGCTCATAATACGCCGCTGCTTGTGCTATACGGTTCAAATATGGGAACAGCTGAAGGAACGGCGCGTGATTTAGCAGATATTGCAATGAGCAAAGGATTTGCACCGCAGGTCGCAACGCTTGATTCACACGCCGGAAATCTTCCGCGCGAAGGAGCTGTATTAATTGTAACGGCGTCTTATAACGGTCATCCGCCTGATAACGCAAAGCAATTTGTCGACTGGTTAGACCAAGCGTCTGCTGATGAAGTAAAAGGCGTTCGCTACTCCGTATTTGGATGCGGCGATAAAAACTGGGCTACTACGTATCAAAAAGTGCCTGCTTTTATCGATGAAACGCTTGCCGCTAAAGGGGCAGAAAACATCGCTGACCGCGGTGAAGCAGATGCAAGCGACGACTTTGAAGGCACATATGAAGAATGGCGTGAACATATGTGGAGTGACGTAGCAGCCTACTTTAACCTCGACATTGAAAACAGTGAAGATAATAAATCTACTCTTTCACTTCAATTTGTCGACAGCGCCGCGGATATGCCGCTTGCGAAAATGCACGGTGCGTTTTCAACGAACGTCGTAGCAAGCAAAGAACTTCAACAGCCAGGCAGTGCACGAAGCACGCGACATCTTGAAATTGAACTTCCAAAAGAAGCTTCTTATCAAGAAGGAGATCATTTAGGTGTTATTCCTCGCAACTATGAAGGAATAGTAAACCGTGTAACAGCAAGGTTCGGCCTAGATGCATCACAGCAAATCCGTCTGGAAGCAGAAGAAGAAAAATTAGCTCATTTGCCACTCGCTAAAACAGTATCCGTAGAAGAGCTTCTGCAATACGTGGAGCTTCAAGATCCTGTTACGCGCACGCAGCTTCGCGCAATGGCTGCTAAAACGGTCTGCCCGCCGCATAAAGTAGAGCTTGAAGCCTTGCTTGAAAAGCAAGCCTACAAAGAACAAGTGCTGGCAAAACGTTTAACAATGCTTGAACTGCTTGAAAAATACCCGGCGTGTGAAATGAAATTCAGCGAATTTATCGCCCTTCTGCCAAGCATACGCCCGCGCTATTACTCGATTTCTTCATCACCTCGTGTCGATGAAAAACAAGCAAGCATCACGGTCAGCGTTGTCTCAGGAGAAGCGTGGAGCGGATATGGAGAATATAAAGGAATTGCGTCGAACTATCTTGCCGAGCTGCAAGAAGGAGATACGATTACGTGCTTTATTTCCACACCGCAGTCAGAATTTACGCTGCCAAAAGACCCTGAAACGCCGCTTATCATGGTCGGACCGGGAACAGGCGTCGCGCCGTTTAGAGGCTTTGTGCAGGCGCGCAAACAGCTAAAAGAACAAGGACAGTCACTTGGAGAAGCACATTTATACTTCGGCTGCCGTTCACCTCATGAAGACTATCTGTATCAAGAAGAGCTTGAAAACGCCCAAAGCGAAGGCATCATTACGCTTCATACCGCTTTTTCTCGCATGCCAAATCAGCCGAAAACATACGTTCAGCACGTAATGGAACAAGACGGCAAGAAATTGATTGAACTTCTTGATCAAGGAGCGCACTTCTATATTTGCGGAGACGGAAGCCAAATGGCACCTGCCGTTGAAGCAACGCTTATGAAAAGCTATGCTGACGTTCACCAAGTGAGTGAAGCAGACGCTCGCTTATGGCTGCAGCAGCTAGAAGAAAAAGGCCGATACGCAAAAGACGTGTGGGCTGGGTAA
